# Awareness of Vital Complications and Associated Factors Among Type 2 Diabetic Patients in Al‐Hudaydah, Yemen: A Cross‐Sectional Study

**DOI:** 10.1155/bmri/3230665

**Published:** 2026-01-24

**Authors:** Khaled Alselwy, Mogeeb Saeed

**Affiliations:** ^1^ Department of Community Medicine, Faculty of Medical and Health Sciences, Hodeida University, Al-Hudaydah, Yemen, hoduniv.edu.ye; ^2^ Department of Medical Laboratories, Medical Microbiology, Hodeida University, Al-Hudaydah, Yemen, hoduniv.edu.ye

**Keywords:** awareness, complications of diabetes, eye examination, hypoglycemia, renal function testing

## Abstract

**Background:**

Yemen ranks 120th in diabetes‐related mortality in 2020, with a mortality rate of 15.42 per 100,000 people. Awareness and early detection of diabetic complications are vital.

**Objective:**

This study is aimed at assessing the awareness of diabetes complications, specifically retinopathy, nephropathy, and hypoglycemia symptoms and associated factors, among patients with Type 2 diabetes mellitus in Al‐Hudaydah, Yemen, in order to reduce diabetes‐related morbidity and mortality.

**Methods:**

A cross‐sectional study was conducted between January and June 2022; 900 randomly selected participants were involved. A prevalidated questionnaire was utilized to assess awareness of diabetic complications. Statistical analyses were performed to identify correlations between demographic factors and awareness levels.

**Results:**

Of 900 patients (mean age 47.6, mostly male), 60.8% understood common health issues and the need for annual care (*p* < 0.001). However, significant awareness gaps were identified: 34.0% were unaware of hypoglycemia symptoms, 40.3% did not recognize the importance of renal testing, and 40.2% lacked awareness of eye examinations. Longer disease duration (> 6 years) was associated with lower awareness of hypoglycemia symptoms (OR = 0.84, 95% CI 0.72–0.98, *p* = 0.025). Females had lower awareness of renal testing (OR = 0.675, 95% CI 0.56–0.82, *p* < 0.01). Higher education doubled awareness (OR = 2.03–2.13, 95% CI 1.50–2.50, *p* < 0.01).

**Conclusion:**

The study highlights significant gaps in awareness of diabetic complications, particularly hypoglycemia symptoms, renal testing importance, and eye examinations. Younger age and higher education levels were positively associated with awareness, while longer disease duration and female gender were negatively correlated. Targeted educational interventions are essential, especially for older populations and those with lower educational attainment, to enhance diabetes self‐care and improve health outcomes.

## 1. Introduction

Diabetes mellitus (DM) is a chronic inflammatory disease that leads to severe and even fatal complications. It is a significant public health challenge in Yemen, with a mortality rate of 15.42 per 100,000 people in 2020, ranking Yemen 120th globally for diabetes‐related mortality [1]. Common complications of Type 2 diabetes mellitus (T2DM) include diabetic nephropathy, acute coronary syndrome, retinopathy leading to vision impairment, cataracts, and myocardial infarction. These complications often develop gradually during the undiagnosed period preceding diagnosis owing to the inability of the body to control the disease process properly [2–7].

Awareness and early detection of diabetic complications are vital to reduce this alarming burden. Previous studies in Yemen have reported low awareness among T2DM patients regarding potential complications, especially cardiovascular risks [8–10]. Understanding hypoglycemia symptoms can vary depending on individual treatment regimens and health literacy levels [11, 12]. This study is aimed at assessing the awareness of diabetes complications, especially retinopathy, nephropathy, and hypoglycemia symptoms, and associated factors among patients with T2DM attending state‐run hospitals and clinics in Al‐Hudaydah, Yemen. The primary goal is to help reduce diabetes‐related morbidity and mortality in the country by improving patient awareness and guiding targeted educational programs.

## 2. Materials and Methods

### 2.1. Study Design and Location

We conducted a cross‐sectional study between January and June 2022 among T2DM outpatients attending state‐run hospitals and clinics in Al‐Hudaydah City, Yemen.

#### 2.1.1. Participants

The study included T2DM patients aged 18 years and older who had been receiving treatment for at least 1 year at participating hospitals and clinics.

#### 2.1.2. Inclusion and Exclusion Criteria

We included all patients with T2DM who had been on medication for more than 1 year and were 18 years old.

We excluded patients with T2DM who had been receiving treatment for less than 1 year, who were seriously ill, and health professionals.

### 2.2. Sample Size Determination and Sampling Techniques

The study population consisted of outpatients with T2DM who attended public hospitals and clinics in Al‐Hudaydah City (three districts: northern, central, and southern) during the study period.

We used stratified random sampling to select participants from each district to guarantee representation. The population of each district was divided into age groups (< 48, 48–60, and > 60 years) and sex (male and female).

A sample of 300 participants was randomly selected from each district using computer‐generated random numbers from each stratum (sex and age group), resulting in 900 participants from the three districts.

The required sample size was calculated as 900 using a single population proportion formula with the following assumptions: 50% proportion of patients with adequate awareness (*p*), 95% confidence interval (CI), and 3% margin of error.

### 2.3. The Study’s Variables

The dependent variable was the awareness of diabetes‐related complications. The independent variables included age, sex, educational status, and diabetes (mean duration of diabetes).

### 2.4. Measures

We used a prevalidated 5‐point Likert scale questionnaire from previous local studies [13] to measure awareness of retinopathy, nephropathy, and hypoglycemia symptoms. The knowledge score ranged from 0 to 8, with a median of 4 (IQR: 2–6). Values above the median were defined as a good level of knowledge. To enhance the validity of the questionnaire, we conducted a pilot study with 90 T2DM patients excluded from the main sample. The pilot study helped refine the survey instrument and ensure the questions were clear, comprehensive, and appropriately measured the desired constructs.

### 2.5. Data Quality Management

Data quality was ensured by cross‐checking 10% of the questionnaire entries for accuracy and consistency. The questionnaire was also pretested on 90 T2DM patients to check for validity.

### 2.6. Statistical Analysis

We perform univariate and multivariate logistic regression analyses using SPSS Version 26 at a significance level of *p* < 0.05. We used univariate analysis to assess the associations between awareness levels (dependent variable) and each independent variable individually (age, sex, education, income, and diabetes duration). Multivariate analysis simultaneously assessed the associations between awareness levels and all independent variables, while controlling for potential confounding factors.

Odds ratios (ORs) and 95% CIs were calculated using the univariate and multivariate models. Assumptions for logistic regression, including the absence of multicollinearity, were checked before analysis.

## 3. Results

### 3.1. Sociodemographic Characteristics

Table 1 represents the sociodemographic characteristics of the 900 surveyed T2DM patients. The mean age was 47.6 years, with the majority being male (66.7%, *n* = 654). Education level was 40.3% (*n* = 396), and the remaining participants had either no formal education or had only completed primary education.

**Table 1:** Characteristics of Type 2 diabetes.

**Patient variable**	**Result**
Age (years)	47.64 ± 15.81
The mean duration of disease (years)	6.24
Sex	
Male	654 (72.7%)
Female	246 (27.3%)
Level of education	
Uneducated	396 (44.0%)
Primary	201 (22.3%)
Secondary	163 (18.1%)
University	140 (15.6%)

### 3.2. Awareness of Diabetic Complications

Table 2 represents the level of awareness among the surveyed patients. A significant percentage (60.8%, *n* = 548) of participants demonstrated a satisfactory level of knowledge (*p* < 0.001).

**Table 2:** Awareness of common diabetic complications.

**Statement**	**Yes,** **n** **(%)**	**No,** **n** **(%)**
Knowledge of the symptoms of hypoglycemia	594 (66.0%)	306 (34.0%)
Awareness of the need for renal tests	537 (59.7%)	363 (40.3%)
Awareness of the need to consult an ophthalmologist	538 (59.8%)	362 (40.2%)

### 3.3. Univariate Analysis of Factors Associated With Awareness

Table 3 represents the results of the univariate analysis, revealing the factors associated with awareness of specific diabetic complications. It was discovered that 34.0% of the participants (*n* = 306) were unaware of hypoglycemia symptoms (OR = 0.60, 95% CI 0.45–0.79, *p* = 0.001). Additionally, 40.3% (*n* = 363) were unaware of the importance of renal testing (OR = 0.71, 95% CI 0.55–0.92, *p* = 0.008), and 40.2% (*n* = 362) did not comprehend the rationale behind eye examinations (OR = 0.71, 95% CI 0.55–0.91, *p* = 0.009). Younger patients (< 48 years) demonstrated higher odds of being aware of eye examinations (OR = 1.37, 95% CI 1.01–1.87, *p* < 0.05).

**Table 3:** Factors associated with awareness of diabetes mellitus complications (univariate analysis).

**Factor (lack of awareness of)**	**Odds ratio (OR)**	**Confidence interval (CI)**	**p** **value**
Hypoglycemia symptoms	0.60	0.45–0.79	0.001
Lack of awareness of renal testing	0.71	0.55–0.92	0.008
Lack of awareness of eye exam rationale	0.71	0.55–0.91	0.009
Younger age (< 48 years) and eye exam awareness	1.37	1.01–1.87	< 0.05

### 3.4. Multivariable Analysis of Factors Associated With Awareness

Table 4 representing the adjusted ORs for the multivariable analysis found that younger patients (< 48 years) demonstrated higher odds of being aware of hypoglycemia symptoms (OR 1.41, CI 1.02–1.95, *p* < 0.05) or (OR 1.37, CI 1.01–1.87) and eye examinations (OR 1.37, 95% CI 1.01–1.87, *p* < 0.05).

**Table 4:** Factors associated with awareness (multivariate analysis).

**Factor**	**Odds ratio**	**Confidence interval**	**p** **value**
	**(OR)**	**(CI)**	
Younger age (< 48 years) and hypoglycemia awareness	1.41	1.02–1.95	< 0.05
Younger age (< 48 years) and eye exam awareness	1.37	1.01–1.87	< 0.05
Diabetes duration > 6 years and hypoglycemia awareness	0.84	0.72–0.98	0.025
Female gender and renal awareness	0.675	0.56–0.82	≤ 0.01
Education beyond the primary level and general awareness	2.03–2.13	1.50–2.50	< 0.001

The mean duration of the disease did not show statistical significance with awareness, except for those with a longer duration (> 6 years) who showed lower awareness of hypoglycemia (OR 0.84, 95% CI 0.72–0.98, *p* = 0.025). Females showed 32.5% lower odds of being aware of the importance of renal testing compared to males (OR 0.675, 95% CI 0.55–0.83, *p* < 0.01). Furthermore, education beyond the primary level doubled the odds of awareness (OR 2.03–2.13, 95% CI 1.50–2.50, *p* < 0.01).

## 4. Discussion

The findings of this study reveal critical gaps in the awareness of diabetic complications, particularly concerning nephropathy, retinopathy, and hypoglycemia symptoms. Notably, the lack of awareness identified in this research underscores a significant public health concern in Yemen, where diabetes poses a growing threat to health outcomes.

Currently, there is limited local literature addressing awareness levels among T2DM patients in Yemen. This lack of awareness aligns with the sole local study, which reported that approximately 59% of participants had heard about diabetic retinopathy [13]. Low awareness levels in both studies underscore a concerning trend among T2DM patients in Yemen, indicating a broader issue in diabetes management and education.

The lack of extensive local studies complicates the comparison and contextualization of these findings within a broader framework. However, what is evident is that the low awareness levels among T2DM patients in Yemen may contribute to increased morbidity and mortality associated with diabetes‐related complications. This knowledge gap can hinder effective self‐management and prompt healthcare interventions, potentially exacerbating the burden of diabetes in the region.

Given the critical role that awareness plays in the prevention and management of diabetic complications, this study serves as a call to action for both healthcare providers and policymakers. Further research is urgently needed to explore the factors influencing awareness and implement targeted educational interventions to bridge knowledge gaps. By doing so, we can hope to improve health outcomes for individuals living with diabetes in Yemen.

The demographic disparities identified in this study, where younger age and higher education levels positively influenced awareness, while longer disease duration and being female were negatively associated, are consistent with findings from regional and global studies [14–20]. For instance, a study from Italy revealed that female sex, lower income, and lower educational level were associated with worse glycemic control in patients with T2DM [21]. Similarly, research in China found that low education level, rural residence, and older age were independently associated with poor awareness of diabetes‐related complications among patients with T2DM [22]. In India, individuals with low socioeconomic status, lack of education, and residing in rural areas exhibited limited knowledge of diabetes complications [23]. In contrast, a study conducted in Singapore revealed that being female, having a higher BMI, having longer diabetes duration, and having a family history of diabetes were linked to better awareness of diabetes complications [24]. However, a recent study from Malaysia reported that only higher education levels were significantly associated with better awareness of T2DM complications [25].

The disparities in our findings regarding gender and disease duration warrant further examination. Specifically, our results demonstrated that a longer disease duration (over 6 years) was associated with lower awareness of hypoglycemia, while females exhibited decreased awareness of renal testing. Other studies have suggested that awareness levels were similar among both genders, with significantly higher awareness noted in patients aged 65 years (*p* = 0.0001) [26].

These contextual variations warrant further exploration. Potential factors, such as cultural influences, healthcare system characteristics, and patient–provider communication patterns, may contribute to the observed differences and should be investigated in future research.

In summary, the results of these studies suggest that older age, lower education level, longer duration of diabetes, and lower income are consistently associated with poorer awareness of the vital complications of T2DM in the Mediterranean and Asian populations. However, there is some disagreement among studies regarding the relationship between sex and knowledge about T2DM complications. Additional research is necessary to validate these results and to develop successful approaches to enhance awareness of T2DM complications among high‐risk populations.

### 4.1. Limitations of the Study

The cross‐sectional design prevents the determination of causality. Unmeasured confounding may be present, and respondents might have answered in a way that aligns with social norms or expectations. Responses may overestimate or underestimate a person’s true knowledge. While we used a consistent sample size of 900 with a low margin of error, and associations were identified, future longitudinal research could provide stronger evidence.

## 5. Conclusion

This study indicates a significant disparity in the awareness of diabetic complications among T2DM patients, with notable deficiencies in understanding hypoglycemia symptoms and the criticality of regular kidney function tests and eye examinations. These findings underscore the demographic divide. Age and educational attainment substantially influence patient awareness and comprehension. As younger patients and those with a higher level of education demonstrate considerably better awareness, targeted educational interventions tailored to older patients and those with less formal education are imperative. This study serves as a clarion call for the healthcare community to prioritize patient education on diabetes management, focusing on bridging the knowledge gap to prevent complications and improve health outcomes among T2DM patients.

Further research into effective educational tools and strategies is crucial to address these disparities and enhance the quality of life of individuals with T2DM. While most understood major complications, knowledge differed by demographics. Partnering with community health workers using social media to target at‐risk groups could optimize outcomes.

### 5.1. Implications for Practice and Research

Targeted education based on the profiles identified here could help optimize patient outcomes locally. Community health workers and social media may effectively reach groups that lack knowledge (older patients and those with less schooling groups). Longitudinal studies exploring how to sustain retention over time are also warranted. Overall, cultural and health system influences on awareness warrant deeper exploration. In conclusion, tailored initiatives addressing variability based on targeted groups can strengthen prevention efforts to curb increasing mortality in Yemen. It remains critical to address limitations through well‐designed, population‐representative research.

## Ethics Statement

Ethical approval has been obtained from the Health Ethical Research Committee (HERC) at the Hodeidah Faculty of Medicine. As this study is neither harmful nor experimental, and no laboratory animals were involved, the decision was made to ethically approve this study (Number 108‐2020). Additionally, the study was conducted following the Helsinki Protocol. We also obtained verbal informed consent from each participant in the study. Confidentiality and privacy during the collection of personal data were guaranteed.

## Conflicts of Interest

The authors declare no conflicts of interest.

## Funding

No funding was received for this manuscript.

## Data Availability

The dataset supporting the conclusions of this article consists of the summarized data presented in the tables within the manuscript. Additional data (in Excel format) used for the statistical analysis may be available from the corresponding author upon reasonable request.
